# Tumor-Infiltrating Lymphocytes as Predictors of Response to Neoadjuvant Chemotherapy in Breast Cancer: Added Value of Morphological Characterization Beyond Quantification

**DOI:** 10.3390/cancers18132065

**Published:** 2026-06-25

**Authors:** Juan Azcárate, Anna Petit, Teresa Soler-Monsó, Eugenia Quirós, Andrea Vethencourt, Agostina Stradella, Amparo García-Tejedor, Maria Jesús Pla-Farnos, Héctor Pérez-Montero, Anna Gumà, Raúl Ortega, Diana Pérez, Cristina Capó, Mar Varela, Luis M. Molinos-Albert, María del Rosario Taco-Sánchez, Esther Guerra, Jan Bosch-Schips, August Vidal, Evelyn Martínez-Pérez, Sonia Pernas, Miguel Gil-Gil, Catalina Falo

**Affiliations:** 1 Multidisciplinary Breast Cancer Unit, Department of Pathology, Hospital Universitari Bellvitge, 08907 Barcelona, Spain; ampetit.lleida.ics@gencat.cat (A.P.); tersoler@hotmail.com (T.S.-M.); equiros@bellvitgehospital.cat (E.Q.); mvarela@iconcologia.net (M.V.); rtaco@bellvitgehospital.cat (M.d.R.T.-S.); meguerra@clinic.cat (E.G.); jan.bosch@vallhebron.cat (J.B.-S.); avidal@bellvitgehospital.cat (A.V.); 2 Instituto de Investigación Biomédica de Bellvitge (IDIBELL), 08908 Barcelona, Spain; acvethencourt@iconcologia.net (A.V.); astradella@iconcologia.net (A.S.); agarciat@bellvitgehospital.cat (A.G.-T.); mjpla@bellvitgehospital.cat (M.J.P.-F.); hpmontero@iconcologia.net (H.P.-M.); anna.guma@bellvitgehospital.cat (A.G.); rortega@bellvitgehospital.cat (R.O.); emperez@iconcologia.net (E.M.-P.); spernas@iconcologia.net (S.P.); mgilgil@iconcologia.net (M.G.-G.); cfalo@iconcologia.net (C.F.); 3 Facultat de Medicina i Ciències de la Salut, Universitat de Barcelona, 08007 Barcelona, Spain; 4 Department of Pathology, Hospital Universitari Arnau de Vilanova, 25198 Lleida, Spain; 5 Multidisciplinary Breast Cancer Unit, Department of Medical Oncology, Institut Català d’Oncologia, 08908 Barcelona, Spain; 6 Multidisciplinary Breast Cancer Unit, Department of Gynecology, Hospital Universitari Bellvitge, 08907 Barcelona, Spain; 7 Multidisciplinary Breast Cancer Unit, Department of Radiation Oncology, Institut Català d’Oncologia, 08908 Barcelona, Spain; 8 Multidisciplinary Breast Cancer Unit, Department of Radiology, Hospital Universitari Bellvitge, 08907 Barcelona, Spain; 9 Multidisciplinary Breast Cancer Unit, Department of Reparative Surgery, Hospital Universitari Bellvitge, 08907 Barcelona, Spain; dperez@bellvitgehospital.cat; 10 Multidisciplinary Breast Cancer Unit, Department of Gynecology, Hospital de Viladecans, 08840 Barcelona, Spain; ccapo.hv@gencat.cat; 11 ISGlobal, 08036 Barcelona, Spain; luis.molinos@isglobal.org; 12 CIBER Enfermedades Infecciosas (CIBERINFEC), Instituto de Salud Carlos III, 28029 Madrid, Spain

**Keywords:** breast cancer, tumor-infiltrating lymphocytes (TILs), neoadjuvant chemotherapy, predictive biomarkers, tumor microenvironment, plasma cells, heterogeneity, intraepithelial infiltrate, luminal B breast cancer

## Abstract

**Background/Objectives**: Tumor-infiltrating lymphocytes (TILs) are recognized predictors of response to neoadjuvant chemotherapy (NACT) and prognosis in breast cancer, particularly in triple-negative (TN) and HER2-positive subtypes. However, the additional predictive value of morphological features of the inflammatory infiltrate beyond TIL quantification is not fully established. We aimed to assess the predictive value of TILs for response to NACT in breast cancer and to determine whether morphological characteristics of the inflammatory infiltrate enhance predictive accuracy. **Methods:** We analyzed 477 patients with stage II–III breast cancer treated with NACT between 2009 and 2016. Diagnostic core needle biopsies were prospectively re-evaluated. TILs were quantified according to International TILs Working Group recommendations. Morphological features of the infiltrate, including cell composition (lymphocytic vs. plasma cell-rich), heterogeneity, and localization, were evaluated using standardized criteria. Pathologic complete response (pCR) was defined as absence of invasive tumor in the breast and in the axillary lymph nodes (ypT0/Tis ypN0). Univariate and multivariate logistic regression analyses were performed to assess the predictive value of TILs (quantitative and morphological assessment) to achieve pCR for the entire cohort and by surrogate molecular subtype. **Results:** A TIL cutoff of >20% was identified as optimal for predicting pCR. High TILs were significantly associated with high-grade tumors, elevatedKi67, HER2-positive and TN subtypes, presence of plasma cells, and intraepithelial and heterogeneous infiltrates. In the overall cohort, TILs > 20% significantly increased the likelihood of pCR (OR 3.9, 95%IC 2.5–6.0, *p* < 0.001) and was an independent predictor of pCR. A combined variable incorporating TIL level and homogeneity improved predictive performance, with homogeneously high TILs emerging as a strong predictor of pCR (OR 5.521, 95%CI 3.174–9.603, *p* < 0.01). Plasma cell-rich and intraepithelial infiltrates were also associated with higher pCR rates (respectively, OR 2.7, 95%CI 1.5–5.0, *p* = 0.001 and OR 2.8, 95%CI 1.6–5.0, *p* < 0.001). Subtype-specific analyses confirmed the predictive value of TILs in TN tumors, but not in HER2-positive ones. Notably, in luminal B-like tumors, high TILs were the only independent predictor of response (OR 17.982, 95%CI 3.115–103.815, *p* = 0.001). **Conclusions:** TIL assessment on routine H&E-stained biopsies is a robust predictor of response to NACT in breast cancer that is readily available, cost-neutral and does not require additional techniques. Integration of simple morphological features significantly enhances predictive accuracy and may refine treatment stratification, particularly in luminal B-like tumors.

## Simple Summary

Tumor-infiltrating lymphocytes (TILs) are established predictors of response to neoadjuvant chemotherapy (NACT) in breast cancer, though the added value of their morphological features remains unclear. In this study, 477 patients with stage II–III breast cancer treated with NACT were analyzed to evaluate both quantitative and morphological characteristics of inflammatory infiltrates. A TIL threshold of >20% was identified as optimal for predicting pathologic complete response (pCR), with high TILs significantly associated with aggressive tumor features and improved response rates. Incorporating morphological traits enhanced predictive accuracy, with homogeneously high TILs emerging as a strong predictor of pCR. Additional features, such as plasma cell-rich and intraepithelial infiltrates, were also linked to higher TILs and higher response rates. Notably, in luminal B-like tumors, high TILs were the only predictor of pCR. These findings support integrating simple morphological assessment with TIL quantification to improve treatment stratification.

## 1. Introduction

Neoadjuvant chemotherapy (NACT) in breast cancer is a well-established therapeutic approach, particularly for patients with locally advanced or biologically aggressive tumors, as it increases the likelihood of breast-conserving surgery [1]. Among other advantages, it allows assessment of the treatment response and other histological features in the surgical specimen, which may help in selecting additional therapies when necessary. Importantly, the achievement of pathologic complete response (pCR) is associated with better prognosis, including improved disease-free survival and overall survival, particularly in chemo-sensitive tumors such as HER2-positive and triple-negative (TN) subtypes [2,3,4]. Currently, the main predictors of response to NACT include the intrinsic molecular subtype and the proliferative index assessed by Ki67 protein expression [5,6].

In recent years, increasing attention has been directed towards the tumor microenvironment as a source of additional predictive and prognostic biomarkers [7]. Among these, tumor-infiltrating lymphocytes (TILs) have emerged as a key component. TILs are defined as the percentage of mononuclear inflammatory cells (lymphocytes and plasma cells) within the tumor stroma and are assessed according to standardized criteria established by Salgado and the international working group in their 2014 publication [8]. TILs are of interest because, when evaluated in the diagnostic biopsy, high levels have been shown to predict response to NACT and serve as a prognostic factor, particularly in TN and HER2-positive tumors [9,10]. Moreover, in immune checkpoint inhibitor therapies used in TN breast cancer, TILs are considered a predictive factor of response [11,12,13].

Beyond the quantification of TILs, recent studies have investigated the architecture, heterogeneity, spatial distribution, and cellular composition of the accompanying inflammatory infiltrate using a wide range of techniques, including conventional histology, digital image analysis, and immunohistochemical and molecular methods [14]. However, their incremental predictive value beyond TIL quantification remains insufficiently defined, particularly using methods that are easily applicable in routine clinical practice.

The aim of this study was to assess the predictive value of TILs for response to neoadjuvant chemotherapy in a large cohort of breast cancer patients treated at our institution and to evaluate whether the morphological characterization of this infiltrate added predictive value on pCR. As a secondary objective, we explore the relationship between TIL percentage, other morphologic characteristics of the inflammatory infiltrate and clinicopathological variables.

## 2. Materials and Methods

### 2.1. Study Population

This study included all consecutive patients diagnosed with stage II and III breast cancer treated with neoadjuvant chemotherapy between 2009 and 2016 at the Breast Functional Unit (UFM for the acronym in Spanish) from the Hospital Universitari de Bellvitge (HUB) and the Institut Català d’Oncologia (ICO, L’Hospitalet de Llobregat, Barcelona, Spain). All patients received primary chemotherapy with a homogeneous schema during that period. It consisted of anthracycline and taxane-based regimens plus trastuzumab in HER2-positive cases. Written informed consent was obtained from all patients and the study received approval from the hospital’s research ethics committee. Clinical outcomes from this cohort were previously reported in June 2024 [3]. Inclusion criteria for the present study required the availability of sufficient histological material in the diagnostic breast core biopsy to enable TIL assessment (quantitative and qualitative analyses). Exclusion criteria included patients with stage-T0 node-positive disease, due to the limitation of assessing TILs according to international guidelines, and patients enrolled in a clinical trial.

### 2.2. Pathology Material

All hematoxylin and eosin (H&E)-stained sections evaluated, as well as all immunohistochemical (IHQ) stains required for the preparation of the diagnostic reports reviewed, were performed on 3 μm, formalin-fixed paraffin-embedded tissue sections. IHC studies with estrogen receptor (ER) (clone 1D5; Dako, Agilent Technologies, Carpinteria, CA, USA), progesterone receptor (PR) (clone PgR 636; Dako, Agilent Technologies, Carpinteria, CA, USA), HER2/Neu (HercepTest; Dako, Agilent Technologies, Carpinteria, CA, USA) and Ki67 (clone MIB-1; Dako, Agilent Technologies, Carpinteria, CA, USA) were performed on an “Autostainer Link48” (Dako, Agilent Technologies, Santa Clara, CA, USA). HER2 in situ hybridization was performed with a HER2 FISH pharmaDx Kit (Dako Denmark A/S, Glostrup, Denmark) when necessary.

All material reviewed was retrieved from the archives of the HUB Department of Pathology.

### 2.3. Pathological Assessment and Data Collection

Diagnostic core needle biopsies (CNBs) obtained prior to initiation of chemotherapy were re-analyzed. A prospective morphological description of the inflammatory infiltrate was performed based on the H&E-stained slides. To ensure consistency, the first 100 biopsies were jointly evaluated by three experienced breast pathology specialists (JA, AP, TSM) to harmonize scoring criteria. The remaining samples were assessed by a single pathologist (JA). All evaluations were performed blinded to clinicopathological characteristics and treatment response.

In addition to quantifying TILs according to the recommendations of the International Immuno-Oncology Biomarker Working Group [15], we evaluated other characteristics of the infiltrate: cell type (lymphocytic, plasma cell-rich, or mixed), heterogeneity, and localization (stromal, intraepithelial, or mixed). For these additional features, we established standardized, easily applicable criteria. The cell type was classified as “plasma cell-rich” (PC) when plasma cells accounted for more than 50% of the infiltrate; as “lymphocyte and plasma cell” (L&PC) when plasma cells were conspicuous but did not exceed half of the infiltrate; and as “lymphocytic” in all other cases.

Heterogeneity was defined as a >30% difference in TIL percentage among the evaluated fields (minimum of 6 fields at 200–400× magnification). Regarding localization, the infiltrate was classified as “stromal” when it was found exclusively or almost exclusively within the stromal compartment, and as “intraepithelial/stromal” (IE&S) when it was present within the epithelial tumor compartment or both epithelial and stromal compartments. Additionally, a composite variable combining heterogeneity/homogeneity and TIL levels (>20% vs. ≤20%) was generated, resulting in four categories: homogeneously high, homogeneously low, heterogeneously high, and heterogeneously low.

Histologic tumor characteristics retrieved from pathology reports included the WHO histologic type [16], histologic grade according to the Nottingham Histologic Score system [17], cases with Ki67 ≥ 30%, and surrogate molecular subtype classification based on the St. Gallen 2013 criteria [1]: LuminalA (LA), LuminalBHER2 negative (LB), LuminalBHER2 positive (HER2+HR+), HER2 Enriched (HER2+HR−) and Triple Negative (TN). Cases with nuclear hormone receptor expression < 10% were considered negative for hormone receptor classification. HER2 positive was defined as IHC 3+ staining or IHC 2+ with gene amplification by in situ hybridization.

Treatment response was calculated using the Residual Cancer Burden (RCB) index [18] with the available online calculator [19]. Data for RCB calculation were collected from pathology reports, with slide review when needed. Pathologic complete response was defined as the absence of invasive carcinoma in the breast and axillary lymph nodes (ypT0/Tis ypN0).

### 2.4. Clinical Data Collection

Clinical variables collected included patient age; clinical stage according to UICC classification [20]; chemotherapy regimen; type of surgical procedure; and dates of treatment initiation, completion, and surgery.

### 2.5. Data Interpretation and Statistical Analysis

A descriptive frequency analysis was performed. Parametric variables are reported as mean and standard deviation, and non-parametric variables as median and range. Qualitative variables are expressed as counts and percentages.

Student’s *t*-test was used to compare continuous variables, and the chi-square or Fisher’s exact test for categorical variables.

A ROC curve analysis was performed to determine the TIL cutoff in our cohort that maximized sensitivity and specificity to predict pCR, which was then used to define a high versus low percentage of TILs.

A chi-square test was used to identify clinical and pathological variables associated with a high percentage of TILs. Logistic regression uni- and multivariate analysis were performed to confirm the results.

Logistic regression analyses were performed to identify factors associated with pCR. Univariate analyses were conducted for each clinicopathological variable (age, TNM stage, Ki67 ≥ 30%, histological type, histological grade, and surrogate molecular subtype), TILs categorized as low or high using the 20% cutoff, and the different morphological characteristics of the infiltrate described above (cell type, heterogeneity, and localization). A multivariate model was subsequently constructed to quantify the additional predictive value of these morphological features beyond that provided by the dichotomized TIL variable, while controlled by all the other clinicopathological variables described above. These analyses were performed for the entire cohort and separately for each surrogate molecular subtype.

Statistical analyses were conducted using IBM SPSS Statistics version 23 (IBM Corp., Armonk, NY, USA). Statistical significance was set at *p* = 0.05.

## 3. Results

### 3.1. Patient and Tumor Characteristics

Of the initial cohort (*n* = 482), 5 cases were excluded due to insufficient histological material, resulting in a final study population of 477 cases. Baseline patient and tumor characteristics are summarized in Table 1. Patients were relatively young (mean age: 50 years), with locally advanced tumors in approximately one-third of cases. Most tumors were of no special type (NST), high grade (grade 2 or 3), and exhibited high proliferative activity (Ki-67 > 30%). Luminal A-like tumors were underrepresented.

### 3.2. Determination of the Optimal TILs Cutoff

**Figure 1:**
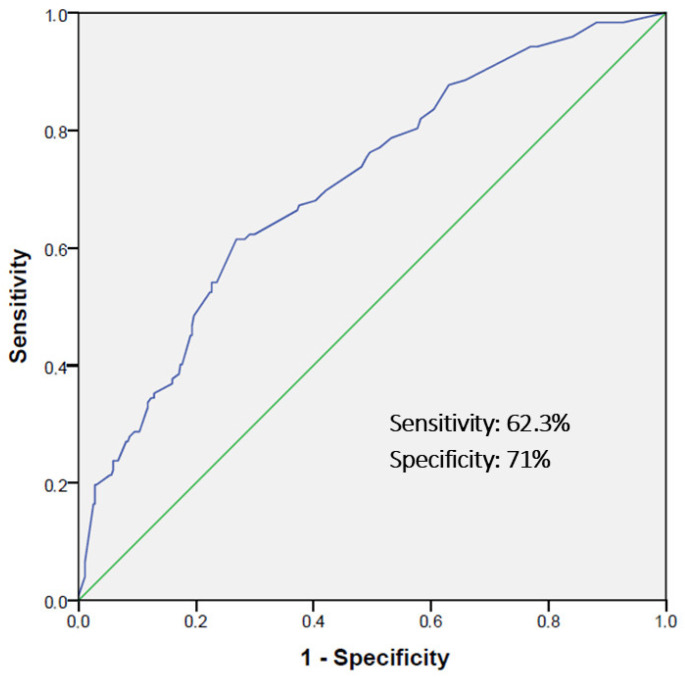
ROC curve including the whole series, used to define the TIL cutoff set at 20%.

One of the objectives of our study was to establish a threshold above which TILs could serve as a predictive biomarker for NACT response. Using a ROC curve, we defined this cutoff as >20%, with a sensitivity of 62.3% and specificity of 71% (see Figure 1). This calculation was performed using the entire cohort and not in each surrogate molecular subtype due to statistical and mathematical requirements.

### 3.3. Quantification and Morphological Assessment of TILs

**Figure 2:**
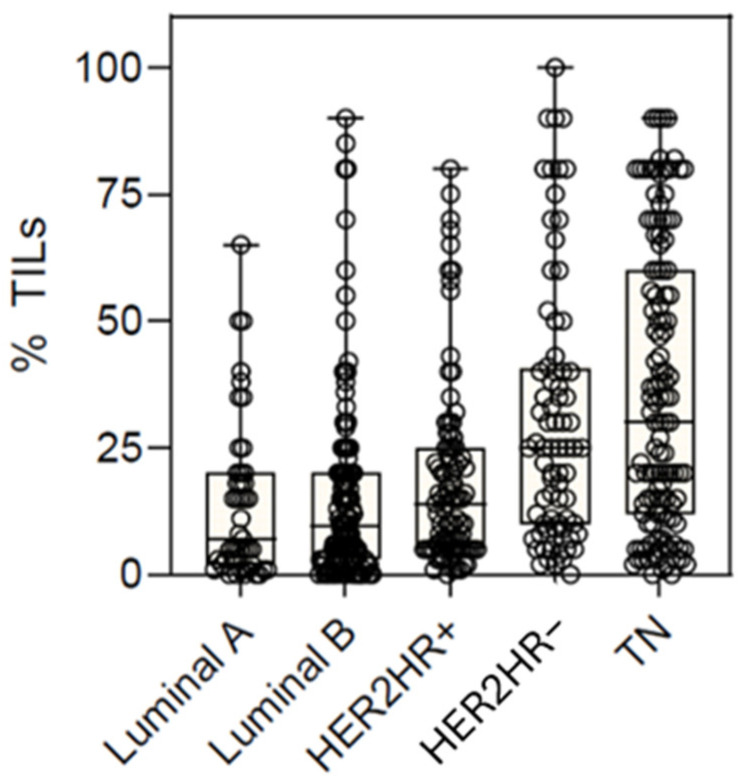
TIL distribution across surrogate molecular subtypes.

TIL characteristics are summarized in Table 2. The median TIL level across the cohort was 15%, with the highest values observed in HER2-positive and TN subtypes. TN and HER2 tumors also displayed greater variability in TIL levels compared with luminal subtypes, which showed a more homogeneous and predominantly low TIL distribution (Figure 2).

A total of 26 cases (5.5%) showed no detectable inflammatory infiltrate, almost exclusively within luminal subtypes. These cases were excluded from subtype-specific analyses of infiltrate shown in Table 2.

**Figure 3:**
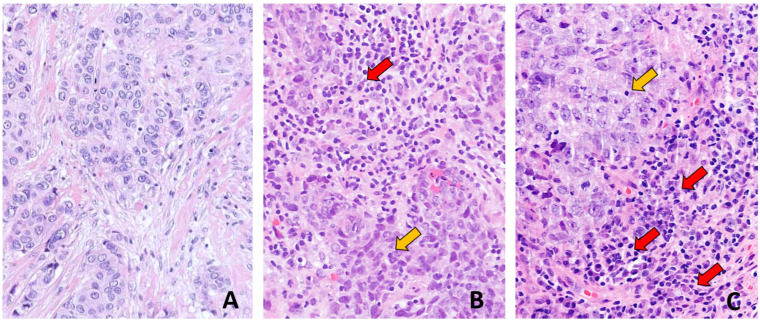
(**A**)**:** Homogeneous low TIL (TIL ≤ 20%), lymphocytic cell type with stromal localization. (H&E, 400x). (**B**): Homogeneous high TIL (TIL > 20%), lymphocyte and plasma cell type (L&PC) with intraepithelial/stromal location (IE&S), (H&E, 400x). (**C**): Homogeneous high TIL (TIL > 20%), plasma cell rich type (PC) with intraepithelial/stromal location (IE&S) (H&E, 400x). Yellow arrows point to the intraepithelial inflammatory component and red arrows point to plasma cells.

**Figure 4:**
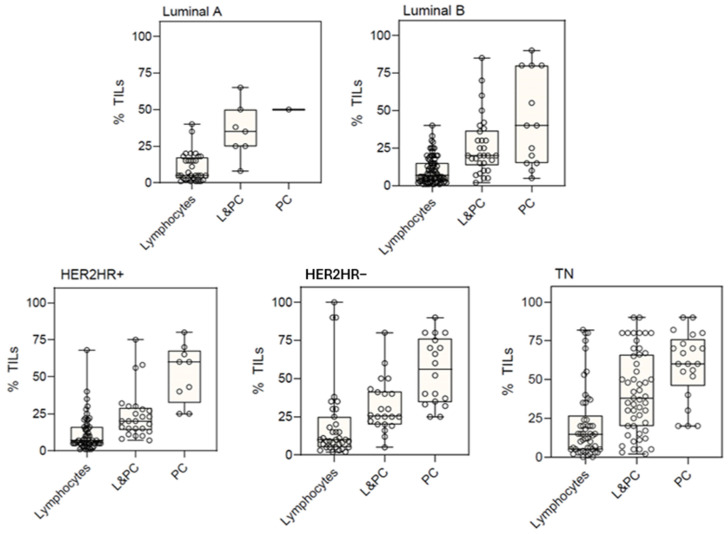
TIL percentage and inflammatory “cell type” composition across surrogate molecular subtypes. Cases with plasma cell-rich (PC) infiltrate showed the highest mean TILs, followed by mixed lymphocyte and plasma cell (L&PC) infiltrates, which displayed more heterogeneity. Cases with purely lymphocytic infiltrates had lower TILs, with a relatively homogeneous distribution and mean TIL values < 20% across all subtypes.

Significant differences among subtypes were found for the variable “cell type”, with HER2 and TN tumors showing a higher proportion of rich PC infiltrates (24% and 17.27%, respectively) or mixed L&PC cell infiltrates (29.3% and 41.8%) (Figure 3 and Figure 4). Regarding infiltrate localization, intraepithelial infiltrate was generally uncommon (Figure 3), being slightly higher in TN tumors (16.4%) but without statistical significance (*p* = 0.445). No significant differences were found in infiltrate homogeneity among subtypes (*p* = 0.066); however, HER2-positive and TN tumors showed a significantly higher proportion of homogeneously high TILs when combining TIL level and heterogeneity (*p* < 0.001).

### 3.4. Variables Associated with TIL > 20%

Clinicopathologic variables significantly associated with high lymphocytic infiltrate (TIL > 20%) included NST histologic subtype, histologic grade 3, high Ki-67, HER2-postitive and TN subtypes (Table 3). Regarding morphological features of the inflammatory infiltrate, high TILs were strongly associated with plasma cell presence, and to a lesser extent, with intraepithelial infiltrate and infiltrate heterogeneity. In multivariate analysis including clinicopathologic and infiltrate variables, the following remained independently significant (from strongest to weakest): plasma cell presence, intraepithelial infiltrate, HER2-postive and TN subtypes, heterogeneous infiltrate, and histologic grade 3. Notably, no lobular carcinomas showed high TIL levels.

### 3.5. Variables Associated with pCR

#### 3.5.1. Complete Series

Of the 477 patients, 120 (25.2%) achieved pCR. Rates varied by subtype: 1 LA (2.2%), 15 LB (10.6%), 26 HER2+HR+ (28.3%), 40 HER2+HR− (52.6%), and 38 TN (31.1%).

Results of the logistic regression univariate and multivariate analyses of variables associated with pCR are summarized in Table 4.

In the univariate model, variables significantly associated with pCR included histologic grade 3, high Ki-67, molecular subtypes HER2+HR+, HER2+HR−, and TN, presence of TIL > 20%, plasma cell presence (PC and L&PC categories), intraepithelial infiltrate, and the combined variable of homogeneously high infiltrate. Patients with homogeneously high TIL levels (>20%) exhibited a stronger association with pCR than those with TIL levels > 20% when infiltrate homogeneity was not taken into account (OR 3.894 vs. 5.521).

In the multivariate analysis, all variables were included except the composite heterogeneity-TILs > 20% variable to avoid redundancy. Histological grade 3, HER2 positivity, high Ki-67 expression, TILs > 20% and homogeneity emerged as independent predictors of pCR, whereas triple-negative tumors showed a borderline significant association. None of the remaining morphological categories showed a significant association with pCR. None of the lobular carcinoma cases achieved pCR.

An alternative multivariate analysis was also performed (available in the Supplementary Material), including all variables. This model yielded very similar results; however, the TILs > 20 variable alone lost statistical significance in favor of the combined heterogeneity-TIL > 20 variable.

#### 3.5.2. Logistic Regression Analysis in Each Surrogate Molecular Subtype

##### Luminal A-like

Only one of the 45 LA cases achieved pCR, precluding statistical analysis of this subgroup (see Table 5). This was a 54-year-old patient with stage IIA invasive ductal carcinoma, grade 3, ER 90%, PR 70% and Ki67 10%. The tumor had TILs of 50%, >50% plasma cells, intraepithelial infiltrate, and was classified as heterogeneously high.

##### Luminal B-like

In LB-like tumors, variables significantly associated with pCR included grade 3, high Ki-67, TIL > 20%, intraepithelial infiltrate, homogeneously high infiltrate and heterogeneously high infiltrate (see Table 6). Notably, pCR rates exceeded 40% in tumors with homogeneously high TILs. L&PC cases showed higher pCR compared with purely lymphocytic infiltrates, though this was not statistically significant. In the multivariate analysis, high TIL was the only independent predictor of pCR (OR 17.982, 95%CI 3.115–103.815, *p* = 0.001).

##### HER2+HR+

Features significantly associated with pCR included grade 3, high Ki-67, plasma cell presence, homogeneously high infiltrate, and intraepithelial infiltrate (see Table 7). In the multivariate analysis, only L&PC infiltrate merged as independent predictor of pCR. Notably, 6 out of 8 cases with intraepithelial infiltrate achieved pCR.

##### HER2+HR−

In this group, pCR rates were high (>50%) across all categories, regardless of the histological characteristics (see Table 8). Cases with high TIL showed higher pCR (55.8% vs. 48.5%), though this was not statistically significant (*p* = 0.644). Plasma cell presence and homogeneously high infiltrate were associated with even higher pCR (>60%). In multivariate analysis, plasma cell presence doubled the probability of pCR, though without statistical significance.

##### Triple Negative

TN patients <50 years achieved higher pCR rates. Other classic predictors associated with pCR were grade 3 and high Ki-67 (see Table 9). TILs retained statistical significance, both as a single variable and when combined with homogeneity. Cases with plasma cells and those with intraepithelial infiltrate achieved higher pCR rates, exceeding 33% and 45%, respectively. In the multivariate analysis, younger age and high TILs were significantly associated with pCR.

## 4. Discussion

The present study demonstrates that tumor-infiltrating lymphocytes (TILs), assessed on routine H&E-stained diagnostic biopsies, are a robust and clinically accessible predictor of response to NACT in breast cancer. Importantly, our findings show that incorporating simple morphological features of the inflammatory infiltrate, particularly the presence of plasma cells (vs. lymphocytes), its location (intraepithelial vs. stromal), and its heterogeneity, may complement the predictive value of TILs beyond their quantitative assessment alone, even if only because their presence is associated with higher TIL levels.

A major strength of this study is that all evaluated TIL variables can be obtained from standard H&E-stained diagnostic biopsies without the need for additional techniques, making this strategy cost-neutral and directly applicable in routine clinical practice.

In this series of breast cancer stage II and III tumors including luminal, HER2 and triple-negative cases, predictors of pCR are consistent with those described in the literature [21,22]: grade 3, Ki-67 > 30%, and HER2-positive or TN subtypes (vs. LB).

Importantly, our study confirms, in line with published data, that TIL quantification in diagnostic biopsies correlates with response to NACT and establishes TILs > 20% as a predictive threshold. Tumors with TIL > 20% showed almost four times higher odds of achieving pCR compared with tumors with low infiltrate. Moreover, when TIL quantification was combined with infiltrate heterogeneity, the predictive performance improved substantially. Tumors with homogeneously high TILs showed the strongest association with response, with the OR increasing to 5.521 in the univariate analysis. This results likely reflect that homogeneous >20% infiltrates have higher TILs than heterogeneous ones (median 65% vs. 36%), and as reported, higher TILs correlate with a higher probability of pCR [10].

One of the objectives of our study was to assess the predictive value of the morphological characteristics of the inflammatory infiltrate. Cases with plasma cell-rich infiltrates showed a 2.7-fold higher probability of achieving pCR compared with cases lacking this feature. Additionally, cases with intraepithelial infiltrates had an OR of 2.8 (95% CI, 1.5–5.0) relative to cases with stromal-only infiltrates in the univariate logistic regression analysis.

In the multivariate analysis, histological grade, Ki67, molecular surrogate subtype, and TILs remained statistically significant independent predictors of pCR. However, the morphological categories lost statistical significance, probably because plasma cell-rich and intraepithelial infiltrates were associated with higher percentages of TILs. Indeed, when we analyzed factors associated with high TIL levels, defined as >20% according to the ROC curve, the variables independently associated with TILs > 20% were Ki-67 > 30%, HER2-positive and triple-negative surrogate molecular subtypes, and specific morphological characteristics of the infiltrate, namely plasma cell-rich, intraepithelial, and heterogeneous patterns. The loss of statistical significance of these morphological categories in the multivariate model is therefore understandable, given their strong association with high TIL levels. Nevertheless, their presence was associated with a substantially greater likelihood of achieving pCR.

Although results for the entire cohort are more robust, our study placed special emphasis on analyzing TIL variables in each surrogate molecular subtype, since, as the literature suggests, the phenotypic and molecular characteristics and the prognostic and predictive role of TILs are unique to each molecular subtype. Consistent with prior literature, our study supports that TN and HER2-postitive tumors are the most immunogenic subtypes, as reflected by higher TIL levels and a greater proportion of tumors with uniformly high TILs [10]. Moreover, we found that these subtypes more frequently exhibited plasma cell-rich infiltrates, supporting emerging evidence that B-cell-related immune responses may play a relevant role in tumor biology and treatment sensitivity (see below).

While most previous studies have focused on the prognostic role of TILs in TN and HER2-positive tumors, given that increased TILs have been correlated with better survival outcomes in these subgroups [9,10], the predictive value of TILs for response to NACT across all molecular subtypes remains less clearly defined (see references below). In this context, our study provides interesting data regarding the predictive value of lymphocytic infiltrates in luminal B-like tumors, which are less frequently reported.

One of the most relevant findings of our study is the identification of a subset of luminal B-like tumors with high (>20%) and homogeneous TILs that exhibit a markedly increased probability of pCR (OR 13.7). Although the composite variable was excluded from the multivariate analysis to avoid redundancy in the statistical model, TILs > 20% considered as a standalone variable remained the only independent predictor of pCR in this subgroup. Although few LB cases had high TILs, this observation is noteworthy given that LB represents the largest proportion of breast cancers and was the most represented subtype in our series. In the literature we find contradictory data, with studies such as those from the German group pointing in that direction [10], while some meta-analyses describe studies in which no significant association is found [23,24,25]. These authors suggest that this lack of association between TILs and pCR in LB may be due to the different cellular composition of these inflammatory infiltrates compared with TN and HER2-postitive tumors [23]. Our findings support the existence of a more immunogenic subset within LB tumors, in which TIL assessment could help identify patients more likely to benefit from NACT. LB is a heterogeneous subtype where endocrine therapy (with or without CDK inhibitors) and chemotherapy yield poor pCR rates [26,27,28,29,30]. Identifying LB tumors that could benefit from primary chemotherapy based on different biomarkers, like easily accessible TIL assessment, would optimize healthcare resources and spare patients unnecessary toxicity.

In HER2-positive tumors, we observed a higher proportion of cases with elevated TILs, consistent with several meta-analyses [23,31]. However, the significant relationship with pCR could not be established, except in HER2+HR+ cases, where an association was seen between pCR and cases with homogeneously high TILs. Nevertheless, interpretation is limited by the small number of cases. The results regarding the relationship of TILs with pCR in HER2-positive tumors have been inconsistent over time. First trials showed a positive association with pCR following trastuzumab-based neoadjuvant chemotherapy [32,33,34,35], whereas in trials adding dual anti-HER2 blockade no association with pCR was found, as observed in the Tryphaena [36], NeoSphere [37], and TRAIN-2 trials [38]. Very interesting results were communicated at San Antonio Breast Cancer Conference 2025 about the CompassHEr2trial, which, with a different cutoff of TILs settled at 10%, obtained similar results as in our study (cutoff of 20% TILs), in the sense that TILs were a predictor of pCR only in the HER2+/ER+ cohort but not in HER2+/ER− [39]. We could hypothesize that when HER2 is dominant—via dual blockade or HR-negativity—other parameters (such as TILs) are relegated to a secondary role, making tumor response largely dependent on the HER2 expression/amplification onco-addictive signal.

In TN cases, our results confirm the well-established predictive role of TILs, with higher TIL levels significantly associated with pCR [23], using the same 20% cutoff determined in previous studies [40,41]. In a systematic review of 92 studies evaluating predictive biomarkers of response to neoadjuvant chemotherapy (NACT) in TN breast cancer, the biomarkers most consistently associated with pCR were high Ki67 expression, PD-L1 expression, and high-density TILs, particularly CD8+ T cells, as well as related immune gene expression signatures [42]. This reinforces the relevance of immune activation in this subtype and aligns with its recognized sensitivity to both chemotherapy and immunotherapy.

Beyond TIL quantification, as previously mentioned, our study highlights the importance of morphological features of the immune infiltrate. Notably, both intraepithelial and plasma cell-rich infiltrates were significantly associated with pCR. However, these associations were not independent of TIL levels, since tumors with plasma cell-rich or intraepithelial infiltrates were also characterized by higher percentages of TILs.

Intraepithelial infiltrate is a known predictor of response to NACT [9,43] and correlates with stromal TIL [9,44]. The International TILs Working Group recommends assessing stromal TILs, not intraepithelial ones, due to parallel distribution, higher abundance, and reproducibility [8], which likely explain the limited literature. Nevertheless, some authors suggest intraepithelial TILs may be biologically more relevant due to their direct contact with tumor cells [8,43]. Khoury et al. [43] showed intraepithelial TILs were independent predictors of pCR in luminal and TN tumors, but not HER2-positive ones. Ruan et al. [41] confirmed this independently in TN tumors. Wu et al. [45] found intraepithelial TILs associated with pCR after NACT across half of analyzed cohorts. In our series, intraepithelial infiltrate was significantly associated with pCR in the whole population and in LB and HER2+HR+ subtypes in univariate analyses, but lost significance in multivariate analysis. Notably in the HER2+HR+ group, 6 out of 8 cases with intraepithelial infiltrate achieved pCR. The interpretation of this parameter was limited by the small number of cases, since only 12% of the cases had intraepithelial infiltrate.

B lymphocytes contribute to antitumor immunity via antigen presentation, antibody production, and modulation of T-cell activity [46]. Plasma cells, as terminally differentiated B cells, are part of this population. Studies on plasma cells largely focus on prognosis [47]. Only one study explored predictive value of plasma cells for NACT response. Sakaguchi et al. [48] found higher plasma cell infiltration in tumors that achieved pCR and a trend toward correlation in HR- tumors, confirmed in a second HR- cohort. Our findings partially aligned: plasma cell presence was significantly associated with pCR in the overall cohort (OR = 2.21, *p* = 0.001), particularly in HER2+HR+ tumors (OR = 3.69, *p* = 0.014). The only LA-like tumor that achieved pCR exhibited a plasma cell-rich infiltrate.

Spatial heterogeneity of TILs and the broader microenvironment influences NACT response and prognosis [47,49,50,51,52,53]. In our study, heterogeneity was defined as >30% TIL variation across fields, and when combined with TIL > 20% it yielded meaningful results. Although an independent association between infiltrate homogeneity and pCR was observed, the absence of a statistically significant association in the univariate analysis warrants a cautious interpretation of these findings.

Several limitations should be acknowledged. First, although the cohort is relatively large, subgroup analyses, particularly within HER2 and TN categories, were limited by sample size. The 20% TIL cutoff identified in this series for predicting pCR may pose a risk of overfitting and should be validated in an independent cohort. The assessment of morphological features such as plasma cell predominance and intraepithelial infiltration was based on semi-quantitative evaluation, which may introduce variability. The use of core needle biopsies precluded the evaluation of features such as the periphery of the tumor or the presence of tertiary lymphoid structures, which may also be relevant components of the immune microenvironment. Finally, more refined characterization of immune cell subpopulations was not possible without immunohistochemical or molecular analyses.

Despite these limitations, our study provides clinically relevant evidence that a more comprehensive evaluation of TILs, integrating both quantitative and morphological parameters, can improve prediction of response to neoadjuvant chemotherapy using widely available tools. Future work could evaluate the use of digital tools for precise quantification in a larger cohort.

## 5. Conclusions

TILs are a robust and clinically relevant predictor of response to neoadjuvant chemotherapy in breast cancer. In our cohort, a threshold of >20% effectively identified tumors with a higher likelihood of achieving pCR. These findings support the routine reporting of TILs in diagnostic biopsy specimens as a simple, cost-neutral biomarker to inform treatment decisions. Importantly, our results demonstrate that incorporating basic morphological features of the immune infiltrate, particularly its cellular composition, intraepithelial location and heterogeneity, provides additional information beyond TIL quantification alone. The identification of tumors with homogeneously high TILs emerged as a strong predictor of response, highlighting the relevance of spatial immune patterns.

From a clinical perspective, these findings are especially meaningful in luminal B-like tumors, a subgroup in which treatment selection remains challenging. The identification of an immunogenic subset with high and homogeneous TILs may help refine patient selection for neoadjuvant chemotherapy and optimize therapeutic strategies.

Morphological characterization of the infiltrate—particularly identifying cases with plasma cells and intraepithelial infiltrate—may further contribute to identifying tumors with high TIL levels and, consequently, a greater likelihood of pCR.

Overall, an integrated evaluation of TILs, combining quantitative and morphological features, offers a pragmatic and scalable approach to improve prediction of treatment response in breast cancer. Further studies are warranted to validate these findings and to better define the biological role of specific immune cell populations and plasma cells.

## Figures and Tables

**Table 1:** Patient characteristics.

	*n* (%)
Complete series	477
**Age**	
Mean	51.06
Standard deviation	12.711
>50	231 (48.4%)
**TNM stage**	
II	327 (68.6%)
III	150 (31.4%)
**Histologic subtype**	
Ductal (NOS)	456 (95.6%)
Lobular	14 (2.9%)
Other	7 (1.5%)
**Surrogate molecular subtype**	
Luminal A	45 (9.4%)
Luminal B	142(29.8%)
HER2+HR+	92 (19.3%)
HER2+HR−	76 (15.9%)
Triple negative	122 (25.6%)
**Histologic grade**	
1	19 (4%)
2	196 (41.1%)
3	260 (54.5%)
**Ki67**	
Median	35
Q1–Q3	25–60
≥30	320 (67.1%)
**TILs %**	
Mean	23.81
Median	15
Q1–Q3	5–35
>20%	182 (38.2%)
**pCR**	
No	357 (74.8%)
Yes	120 (25.2%)

**Table 2:** Characteristics of the inflammatory infiltrate.

TILs	GLOBAL *n* = 477	LA *n* = 45	LB *n* = 142	HER2HR+ *n* = 92	HER2HR− *n* = 76	TN *n* = 122	
Mean	23.81	14.02	15.3	18.83	31.49	36.3	
Median	15	7	9.5	14	25	30	
Q1–Q3	5–35	2–20	3–20	5–25	10–41	12–60	
>20%	182 (38.2%)	9 (20%)	30 (21.1%)	31 (33.7%)	43 (56.6%)	69 (56.6%)	
TIL cases = 0	26 (5.5%)	5 (11.1%)	19 (13.3%)	1 (1.08%)	1 (1.3%)	0 (0%)	
TIL cases > 0	451 (94.5%)	40 (88.9%)	128 (86.7%)	91 (98.92%)	75 (98.7%)	122 (100%)	
**Morphologic characteristics of TIL > 0 cases**							
Cellular type							*p* < 0.00
Lymphocytes	254 (53.2%)	32 (80%)	80 (65%)	57 (62.6%)	35 (46.7%)	50 (41%)	
PC	62 (12.9%)	1 (2.5%)	13 (10.6%)	9 (9.9%)	18 (24%)	21 (17.21%)	
L&PC	135 (28.3%)	7 (17.51%)	30 (24.4%)	25 (27.5%)	22 (29.3%)	51 (41.80%)	
Localization							*p* = 0.445
Stromal	397 (83.2%)	35 (87.5%)	111 (90.2%)	83 (91.2%)	66 (88%)	102 (83.6%)	
IE&S	54 (11.3%)	5 (12.5%)	12 (9.8%)	8 (8.8%)	9 (12%)	20 (16.4%)	
Heterogeneity							*p* = 0.066
Homogeneous	286 (60%)	33 (82.5%)	79 (64.2%)	57 (62.6%)	48 (64%)	69 (56.6%)	
Heterogeneous	165 (34.6%)	7 (17.5%)	44 (35.8%)	34 (37.4%)	27 (36%)	53 (43.4%)	
Heterogeneity-20%							*p* < 0.00
Homogeneous ≤20%	191 (40%)	31 (77.5%)	62 (50.4%)	45 (49.5%)	23 (30.7%)	30 (24.6%)	
Homogeneous >20%	95 (19.9%)	2 (5%)	17 (13.8%)	12 (13.2%)	25 (33.3%)	39 (32%)	
Heterogeneous ≤20%	78 (16.4%)	0 (0%)	31 (25.2%)	15 (16.5%)	9 (12%)	23 (18.9%)	
Heterogeneous >20%	87 (18.2%)	7 (17.5%)	13 (10.6%)	19 (20.9%)	18 (24%)	30 (24.6%)	

LA: Surrogate luminal A; LB: surrogate luminal B; HER2+HR+: Her2 positive and hormone receptor positive; HER2+HR−: Her2 positive and hormone receptor negative; TN: Triple negative; PC: Plasma cell-rich; L&PC: lymphocyte and plasma cell; IE&S: intraepithelial and stromal.

**Table 3:** Clinicopathologic features associated with TIL > 20%.

Global	≤20%	>20%	Univariate			Multivariate		
*n* = 477	*n* = 295	*n* = 182	OR	95%IC	*p*	OR	95%IC	*p*
**Age**								
Mean	50.88	51.35						
Standard deviation	12.4	13.1						
≤50	157 (63.8%)	89 (36.2%)	0.841	0.581–1.218	0.359	0.816	0.472–1.411	0.467
>50	138 (59.7%)	93 (40.3%)	ref					
**TNM Stage**								
II	199 (60.9%)	128 (39.1%)	1.143	0.766–1.707	0.512	1.173	0.661–2.083	0.586
III	96 (64%)	54 (36%)	ref					
**Histologic type**								
Ductal (NOS)	278 (61%)	178 (39%)	ref			ref		
Lobular	14 (100%)	0 (0%)	-			-		
Others	3 (42.9%)	4 (57.1%)	2.08	0.461–9.414	0.341	3.267	0.451–23.652	0.241
**Histologic grade**								
1 and 2	177 (82.3%)	38 (17.7%)	ref			ref		
3	117 (45%)	143 (55%)	5.693	3.714–8.727	0.000	2.120	1.119–4.019	**0.021**
Molecular Subtype *								
Luminal A	36 (80%)	9 (20%)	0.933	0.405–2.149	0.071	3.189	0.891–11.418	0.075
Luminal B	112 (78.9%)	30 (21.1%)	ref			ref		
HER2+HR+	61 (66.3%)	31 (33.7%)	1.897	1.051–3.426	0.034	1.982	0.913–4.302	0.084
HER2+HR−	33 (43.4%)	43 (56.6%)	4.865	2.652–8.924	0.000	3.528	1.566–7.948	0.002
TN	53 (43.4%)	69 (56.6%)	4.86	2.835–8.332	0.000	2.254	1.110–4.577	0.025
**Ki67 > 30%**								
<30%	127 (80.9%)	30 (19.1%)	ref			ref		
≥30%	168 (52.5%)	152 (47.5%)	3.83	2.432–6.033	0.000	1.895	0.925–3.881	0.081
**Cellular type**								
Lymphocytes	209 (82.3%)	45 (17.7%)	ref		0.000	ref		
Plasma cell-rich	8 (12.9%)	54 (87.1%)	31.35	13.95–70.42	0.000	24.445	9.881–60.474	0.000
L&PC	52 (38.5%)	83 (61.5%)	7.413	4.619–11.899	0.000	4.549	2.644–7.829	0.000
**Localization**								
Stromal	263 (66.2%)	134 (33.8%)	ref			ref		
IE&S	6 (11.1%)	48 (88.9%)	15.7.1	6.553–37.621	0.000	9.527	3.485–26.041	0.000
**Heterogeneity**								
Homogeneous	191 (66.8%)	95 (33.2%)	ref			ref		
Heterogeneous	78 (47.3%)	87 (52.7%)	2.243	1.515–3.320	0.000	2.091	1.244–3.516	0.005

HER2+HR+: HER2 positive and hormone receptor positive; HER2+HR−: Her2 positive and hormone receptor negative; TN: Triple negative; L&PC: lymphocyte and plasma cell; IE&S: intraepithelial and stromal; “-”: mathematical error; *: surrogate; “ref”: reference category.

**Table 4:** Clinicopathologic features associated with pCR in the overall cohort.

Global	No pCR	pCR	Univariate			Multivariate		
*n* = 477	*n* = 357	*n* = 120	OR	95%IC	*p*	OR	95%IC	*p*
**Age**								
Mean	51.1	50.9						
Standard deviation	12.5	13.1						
≤50	183 (74.4%)	63 (25.6%)	1.051	0.695–1.590	0.814	1.282	0.787–2.086	0.318
>50	174 (75.3%)	57 (24.7%)	ref			ref		
**TNM Stage**								
II	241 (73.7%)	86 (26.3%)	1.217	0.773–1.918	0.396	1.310	0.764–2.248	0.327
III	116 (77.3%)	34 (22.7%)	ref			ref		
**Histologic grade**								
1 and 2	193 (89.8%)	22 (10.2%)	ref			ref		
3	164 (63.1)	96 (36.9%)	5.135	3.090–8.534	0.000	2.067	1.076–3.817	0.029
Molecular subtype *								
Luminal A	44 (97.8%)	1 (2.2%)	0.192	0.025–1.499	0.116	0.463	0.054–3.966	0.482
Luminal B	127 (89.4%)	15 (10.6%)	ref			ref		
HER2+HR+	66 (71.7%)	26 (28.3%)	3.335	1.654–6.728	0.001	3.235	1.513–6.919	0.002
HER2+HR−	36 (47.4%)	40 (52.6%)	9.407	4.675–18.932	0.000	6.668	3.031–14.719	0.000
TN	84 (68.9%)	38 (31.1%)	3.83	1.983–7.397	0.000	2.026	0.971–4.231	0.060
**Ki67 > 30%**								
<30%	141 (89.8%)	16 (10.2%)	ref			ref		
≥30%	216 (67.5%)	104 (32.5%)	4.243	2.406–7.484	0.000	2.087	1.016–4.288	0.045
**TILs**								
≤20%	250 (84.7%)	45 (15.3%)	ref			ref		
>20%	107 (58.8%)	75 (41.2%)	3.894	2.524–6.007	0.000	2548	1413–4596	0.002
**Cellular type**								
Lymphocytes	206 (81.1%)	48 (18.9%)	ref			ref		
Plasma cell-rich	38 (61.3%)	24 (38.7%)	2.711	1.488–4.938	0.001	0.794	0.365–1.726	0.560
L&PC	89 (65.9%)	46 (34.1%)	2.218	1.380–3.566	0.001	1.247	0.693–2.245	0.462
No infiltrate	24 (92.3%)	2 (7.7%)	0.358	0.082–1.565	0.172	0.860	0.166–4.471	0.858
**Localization**								
Stromal	304 (76.6%)	93 (23.4%)	ref			ref		
IE&S	29 (53.7%)	25 (46.3%)	2.818	1.573–5.049	0.000	-		
No infiltrate	24 (92.3%)	2 (7.7%)	0.272	0.063–1.174	0.081	-		
**Heterogeneity**								
Homogeneous	204 (71.3%)	82 (28.7%)	ref			ref		
Heterogeneous	129 (78.2%)	36 (21.8%)	0.694	0.443–1.088	0.112	0.431	0.255–0.728	0.002
No infiltrate	24 (92.3%)	2 (7.7%)	0.207	0.048–0.897	0.035			
**Heterogeneity-20%**								
Homogeneous ≤20	159 (83.2%)	32 (16.8%)	ref					
Homogeneous >20	45 (47.4%)	50 (52.6%)	5.521	3.174–9.603	0.000			
Heterogeneous ≤20	67 (85.9%)	11 (14.1%)	0.816	0.388–1.713	0.591			
Heterogeneous >20	62 (71.3%)	25 (28.7%)	2.004	1.1–3.65	0.023			
No infiltrate	24 (92.3%)	2 (7.7%)	0.414	0.093–1.840	0.247			

HER2+HR+: Her2 positive and hormone receptor positive; HER2+HR−: Her2 positive and hormone receptor negative; TN: Triple negative; L&PC: lymphocyte and plasma cell; IE&S: intraepithelial and stromal; “-”: mathematical error; *: surrogate; “ref”: reference category.

**Table 5:** Clinicopathologic features associated with pCR in LA-like cases.

Luminal A	No pCR	pCR
*n* = 45	*n* = 44	*n* = 1
**Age**		
≤50	27 (100%)	0 (0%)
>50	17 (94.4%)	1 (5.6%)
**TNM stage**		
II	26 (96.3%)	1 (3.7%)
III	18 (100%)	0 (0%)
**Histologic type**		
Ductal (NOS)	38 (97.4%)	1 (2.6%)
Lobular	4 (100%)	0 (0%)
Others	2 (100%)	0 (0%)
**Histologic grade**		
1 and 2	41 (100%)	0 (0%)
3	3 (75%)	1 (25%)
**Ki67 > 30%**		
<30%	44 (97.8%)	1 (2.2%)
≥30%	0 (0%)	0 (0%)
**TILs**		
≤20%	36 (100%)	0 (0%)
>20%	8 (88.9%)	1 (11.1%)
**Cellular type**		
Lymphocytes	32 (100%)	0 (0%)
Plasma cell-rich	0 (0%)	1 (100%)
L&PC	7 (100%)	0 (0%)
No infiltrate	5 (100)	0 (0%)
**Localization**		
Stromal	35 (100%)	0 (0%)
IE&S	4 (80%)	1 (20%)
No infiltrate	5 (100%)	0 (0%)
**Homogeneity**		
Homogeneous	33 (100%)	0 (0%)
Heterogeneous	6 (85.7%)	1 (14.3%)
No infiltrate	5 (100%)	0 (0%)
**Heterogeneity-20%**		
Homogeneous ≤20	31 (100%)	0 (0%)
Homogeneous >20	2 (100%)	0 (0%)
Heterogeneous ≤20	0 (0%)	0 (0%)
Heterogeneous >20	6 (85.7%)	1 (14.3%)
No infiltrate	5 (100%)	0 (0%)

L&PC: lymphocyte and plasma cell; IE&S: intraepithelial and stromal.

**Table 6:** Clinicopathologic features associated with pCR and univariate and multivariate analysis in LB-like cases.

Luminal B	No pCR	pCR	Univariate			Multivariate		
*n* = 142	*n* = 127	*n* = 15	OR	95%IC	*p*	OR (IC95%)	95%IC	*p*
**Age**								
≤50	73 (89%)	9 (11%)	1.11	0.373–3.304	0.852	0.935	0.245–3.571	0.921
>50	54 (90%)	6 (10%)	ref			ref		
**TNM stage**								
II	84 (87.5%)	12 (12.5%)	2.048	0.548–7.646	0.286	1.132	0.216–5.929	0.884
III	43 (43.5%)	3 (6.5%)	ref			ref		
**Histologic grade**								
1 and 2	83 (95.4%)	4 (4.6%)	ref			ref		
3	44 (80%)	11 (20%)	5.187	1.560–17.246	0.007	3.556	0.795–15.904	0.097
**Ki67 > 30%**								
<30%	44 (97.8%)	1 (2.2%)	ref			ref		
≥30	83 (85.6%)	14 (14.4%)	7.422	0.945–58.313	0.057	2.545	0.251–25.816	0.429
**TILs**								
≤20%	107 (95.5%)	5 (4.5%)	ref			ref		
>20%	20 (66.7%)	10 (33.3%)	10.7	3.305–34.645	0.000	17.982	3.115–103.815	0.001
**Cellular type**								
Lymphocytes	72 (90%)	8 (10%)	ref		0.616	ref		
Plasma cell-rich	12 (92.3%)	1 (7.7%)	0.75	0.086–6.548	0.795	0.066	0.005–0.936	0.045
L&PC	25 (83.3%)	5 (16.7%)	1.8	0.539–6.015	0.34	0.402	0.075–2.159	0.288
No infiltrate	18 (94.7%)	1 (5.3%)	0.5	0.059–4.258	0.526	1.109	0.105–11.684	0.931
**Localization**								
Stromal	102 (91.9%)	9 (8.1%)	ref			ref		
IE&S	7 (58.3%)	5 (41.7%)	8.075	2.131–30.754	0.002	-		
**Heterogeneity**								
Homogeneous	69 (87.3%)	10 (12.7%)	ref			ref		
Heterogeneous	40 (90.9%)	4 (9.1%)	0.690	0.203–2.345	0.552	0.264	0.056–1.259	0.095
No infiltrate	18 (94.7%)	1 (5.3%)	0.383	0.046–3.194	0.375			
**Heterogeneity-20%**								
Homogeneous ≤20	59 (95.2%)	3 (4.8%)	ref					
Homogeneous >20	10 (58.2%)	7 (41.2%)	13.767	3.043–62.285	0.001			
Heterogeneous ≤20	30 (96.8%)	1 (3.2%)	0.656	0.065–6.574	0.72			
Heterogenous >20	10 (76.9%)	2 (23.1%)	5.9	1.041–33.447	0.045			
No infiltrate	18 (94.7%)	1 (5.3%)	1.093	0.107–11.161	0.94			

pCR: pathologic complete response; L&PC: lymphocyte and plasma cell; IE&S: intraepithelial and stromal; “-”: mathematical error; “ref”: reference category.

**Table 7:** Clinicopathologic features associated with pCR and univariate and multivariate analysis in HER2+HR+.

HER2+HR+	No pCR	pCR	Univariate			Multivariate		
*n* = 92	*n* = 66	*n* = 26	OR	95%IC	*p*	OR	95%IC	*p*
**Age**								
≤50	35 (70%)	15 (30%)	1.208	0.483–3.019	0.686	1.090	0.382–3.110	0.872
>50	31 (73.8%)	11 (26.2%)	ref			ref		
**TNM stage**								
II	48 (68.6%)	22 (31.4%)	2.062	0.624–6.815	0.235	2.234	0.581–8.588	0.242
III	18 (81.8%)	4 (18.2%)	ref			ref		
**Histologic grade**								
1 and 2	41 (83.7%)	8 (16.3%)	ref			ref		
3	25 (58.1%)	18 (41.9%)	3.69	1.399–9.734	0.008	2.932	0.902–9.538	0.074
**Ki67 > 30%**								
<30%	31 (86.1%)	5 (13.9%)	ref			ref		
≥30	35 (62.5%)	21 (37.5%)	3.72	1.253–11.048	0.018	2.338	0.647–8.454	0.195
**TILs**								
≤20%	45 (73.8%)	16 (26.2%)	ref			ref		
>20%	21 (67.7%)	10 (32.3%)	1.339	0.521–3.445	0.544	0.654	0.159–2.729	0.565
**Cellular type**								
Lymphocytes	47 (82.5%)	10 (17.5%)	ref			ref		
Plasma cell-rich	5 (55.6%)	4 (44.4%)	3.76	0.855–16.541	0.08	3.256	0.451–23.508	0.242
L&PC	14 (56%)	11 (44%)	3.693	1.3–10.488	0.014	3.952	1.141–13.683	0.030
No infiltrate	0 (0%)	1 (100%)	-			-		
**Localization**								
Stromal	64 (77.1%)	19 (22.9%)	ref			ref		
IE&S	2 (25%)	6 (75%)	10.105	1.883–54.233	0.007	-		
No infiltrate	0 (0%)	1 (1%)	-			-		
**Heterogeneity**								
Homogeneous	39 (68.4%)	18 (31.6%)	ref			ref		
Heterogeneous	27 (79.4%)	7 (20.6%)	0.562	0.206–1.729	0.259	0.316	0.091–1.097	0.070
No infiltrate	0 (0%)	1 (100%)	-					
**Heterogeneity-20%**								
Homogeneous ≤20	34 (75.6%)	11 (24.4%)	ref		0.17			
Homogeneous >20	5 (41.7%)	7 (58.3%)	4.327	1.140–16.425	0.031			
Heterogeneous ≤20	11 (73.3%)	4 (26.7%)	1.124	0.297–4.254	0.863			
Heterogeneous >20	16 (84.2%)	3 (15.8%)	0.58	0.142–2.369	0.448			
No infiltrate	0 (0%)	1 (100%)	-					

pCR: pathologic complete response; L&PC: lymphocytes and plasma cells; IE&S: intraepithelial and stromal: “-”: mathematical error; “ref”: reference category.

**Table 8:** Clinicopathologic features associated with pCR and univariate and multivariate analysis in HER2+HR−.

HER2+HR−	No pCR	pCR	Univariate			Multivariate		
*n* = 76	*n* = 36	*n* = 40	OR	95%IC	*p*	OR	95%IC	*p*
**Age**								
≤50	13 (48.1%)	14 (51.9%)	0.953	0.372–2.440	0.92	0.778	0.272–2.227	0.640
>50	23 (46.9%)	26 (53.1%)	ref			ref		
**TNM stage**								
II	21 (48.8%)	22 (51.2%)	0.813	0.352–2.168	0.77	0.772	0.281–2.119	0.615
III	15 (45.5%)	18 (54.5%)	ref			ref		
**Histologic grade**								
1 and 2	7 (43.8%)	9 (56.3%)	ref			ref		
3	29 (49.2%)	30 (50.8%)	0.805	0.265–2.446	0.702	0.449	0.116–1.744	0.248
**Ki67 > 30%**								
<30%	11 (55%)	9 (45%)	ref			ref		
≥30	25 (44.6%)	31 (55.4%)	1.516	0.543–4.230	0.427	1.984	0.558–7.059	0.290
**TILs**								
≤20%	17 (51.5%)	16 (48.5%)	ref			ref		
>20%	19 (44.2%)	24 (55.8%)	1.342	0.540–3.335	0.526	0.935	0.263–3.321	0.918
**Cellular type**								
Lymphocytes	19 (54.3%)	16 (45.7%)	ref			ref		
Plasma cell-rich	7 (38.9%)	11 (61.1%)	1.866	0.586–5.939	0.291	2.226	0.560–12.670	0.219
L&PC	9 (40.9%)	13 (59.1%)	1.715	0.583–5.047	0.327	2.734	0.715–10.449	0.141
No infiltrate	1 (100%)	0 (0%)	-			-		
**Localization**								
stromal	30 (45.5%)	36 (54.5%)	ref			ref		
IE&S	5 (55.6%)	4 (44.4%)	0.667	0.164–2.707	0.571	-		
No infiltrate	1 (100%)	0 (0%)	-			-		
**Heterogeneity**								
Homogeneous	19 (39.6%)	29 (60.4%)	ref			ref		
Heterogeneous	16 (59.3%)	11 (40.7%)	0.450	0.172–1.178	0.104	0.376	0.146–0.966	0.044
No infiltrate	1 (100%)	0 (0%)	-					
**Heterogeneity-20%**								
Homogeneous ≤20	10 (43.5%)	13 (56.5%)	ref		0.533			
Homogenous >20	9 (36%)	16 (64%)	1.368	0.429–4.364	0.597			
Heterogeneous ≤20	6 (66.7%)	3 (33.3%)	0.385	0.077–1.929	0.245			
Heterogenous >20	10 (55.6%)	8 (44.4%)	0.615	0.178–2.132	0.444			
No infiltrate	1 (100%)	0 (0%)	-					

pCR: pathologic complete response; L&PC: lymphocyte and plasma cell; IE&S: intraepithelial and stromal; “-”: mathematical error; “ref”: reference category.

**Table 9:** Clinicopathologic features associated with pCR and univariate and multivariate analysis in TN.

TN	No pCR	pCR	Univariate			Multivariate		
*n* = 122	*n* = 84	*n* = 38	OR	95%IC	*p*	OR	95%IC	*p*
**Age**								
≤50	35 (58.3%)	25 (41.7%)	2.692	1.212–5.982	0.015	2.495	1.002–6.213	0.049
>50	49 (79%)	13 (21%)	ref			ref		
**TNM stage**								
II	62 (68.1%)	29 (31.9%)	1.143	0.469–2.790	0.768	1.236	0.426–3.587	0.696
III	22 (71%)	9 (29%)	ref			ref		
**Histologic grade**								
2	21 (95.5%)	1 (4.5%)	ref			ref		
3	63 (63.6%)	36 (36.4%)	12	1.549–92.978	0.017	4.485	5.510–39.427	0.176
**Ki67 >30%**								
<30%	11 (100%)	0 (0%)	ref			ref		
≥30	73 (65.8%)	38 (34.2%)	-			-		
**TILs**								
≤20%	45 (84.9%)	8 (15.1%)	ref			ref		
>20%	39 (56.5%)	30 (43.5%)	4.327	1.777–10.537	0.001	5.131	1.576–16.712	0.007
**Cellular type**								
Lymphocytes	36 (72%)	14 (28%)	ref		0.823	ref		
Plasma cell-rich	14 (66.7%)	7 (33.3%)	1.286	0.429–3.852	0.654	0.288	0.068–1.223	0.092
L&PC	34 (66.7%)	17 (33.3%)	1.286	0.550–3.004	0.562	0.630	0.211–1.881	0.408
**Localization**								
stromal	73 (71.6%)	29 (28.4%)	ref			ref		
IE&S	11 (55%)	9 (45%)	2.06	0.773–5.490	0.149	-		
**Heterogeneity**								
Homogeneous	44 (63.8%)	25 (36.2%)	ref			ref		
Heterogeneous	40 (75.5%)	13 (24.5%)	0.572	0.258–1.267	0.169	0.376	0.146–0.966	0.042
**Heterogeneity-20%**								
homogeneous ≤20	25 (83.3%)	5 (16.7%)	ref		0.005			
homogeneous >20	19 (48.7%)	20 (51.3%)	5.263	1.671–16.577	0.005			
heterogeneous ≤20	20 (87%)	3 (13%)	0.75	0.160–3.524	0.716			
heterogeneous >20	20(66.7%)	10 (33.3%)	2.5	0.735–8.502	0.142			

pCR: pathologic complete response; L&PC: lymphocyte and plasma cell; IE&S: intraepithelial and stromal; “-”: mathematical error; “ref”: reference category.

## Data Availability

The data can be shared on request.

## Supplementary Materials

The following supporting information can be downloaded at: https://www.mdpi.com/article/10.3390/cancers18132065/s1, Table S1: Additional multivariate analysis of all clinicopathologic features associated to pCR in the overall cohort.

## Abbreviations

AJCC	American Joint Committee on Cancer
CI	Confidence interval
CNBs	Diagnostic core needle biopsies
H&E	Hematoxylin and eosin
HER2+HR−	HER2 enriched
HER2+HR+	Luminal B HER2 positive
HUB	Hospital Universitari de Bellvitge
ICO	Institut Català d’Oncologia
IE&S	Intraepithelial and stromal
IHQ	Immunohistochemistry
L&PC	Lymphocyte and plasma cell
LA	Luminal A
LB	Luminal B HER2 negative
NACT	Neoadjuvant chemotherapy (NACT)
NST	Of no special type
OR	Odds ratio
PC	Plasma cell-rich
pCR	Pathologic complete response
RCB	Residual Cancer Burden
SD	Standard deviation
TILs	Tumor-infiltrating lymphocytes
TN	Triple negative
UFM	Breast Functional Unit
