# Supplementary material for: Tumor-Infiltrating Lymphocytes as Predictors of Response to Neoadjuvant Chemotherapy in Breast Cancer: Added Value of Morphological Characterization Beyond Quantification
Source: Cancers (Basel). 2026 Jun 25;18(13):2065. doi: 10.3390/cancers18132065 (PMC13360514; doi:10.3390/cancers18132065)
Supplement: Supplementary file 1 [file cancers-18-02065-s001.zip › cancers-4328334-supplementary.pdf]

**SUPLEMENTARY MATERIAL:**

Tables S1.1 (coding of variables) and S1.2 (multivariate analysis): Additional multivariate analysis of all clinicopathologic features associated to pCR in the overall cohort.

Codificaciones de variables categóricas

| | | Frecuencia | Codificación de parámetro | | | |
|---|---|---|---|---|---|---|
| | | | (1) | (2) | (3) | (4) |
| Combinada homogeneidadTIls | homogeni <= 20%TILs | 190 | ,000 | ,000 | ,000 | ,000 |
| | Homogeni >20% TILs | 94 | 1,000 | ,000 | ,000 | ,000 |
| | Heterogeni <=20%TILs | 78 | ,000 | 1,000 | ,000 | ,000 |
| | Heterogeni>20%TILs | 87 | ,000 | ,000 | 1,000 | ,000 |
| | No infiltrat | 26 | ,000 | ,000 | ,000 | 1,000 |
| Molecular sutipo | Luminal A | 45 | 1,000 | ,000 | ,000 | ,000 |
| | Luminal B Her-2 neg | 142 | ,000 | ,000 | ,000 | ,000 |
| | Luminal B Her-2 posit | 92 | ,000 | 1,000 | ,000 | ,000 |
| | Her-2 enriched | 75 | ,000 | ,000 | 1,000 | ,000 |
| | Triple negativo | 121 | ,000 | ,000 | ,000 | 1,000 |
| Tipo celularidad infiltrado | Limfocitos | 253 | ,000 | ,000 | ,000 | |
| | Plasmaticas | 61 | 1,000 | ,000 | ,000 | |
| | Limfocitos+plasmaticas | 135 | ,000 | 1,000 | ,000 | |
| | No infiltrado | 26 | ,000 | ,000 | 1,000 | |
| Homogeneidad_cat3 | Homogeneo | 284 | ,000 | ,000 | | |
| | Heterogeneo | 165 | 1,000 | ,000 | | |
| | No infiltrado | 26 | ,000 | 1,000 | | |
| estromal_intraepitelial-cat3 | estromal | 395 | ,000 | ,000 | | |
| | intraepitelial con o sin estromal | 54 | 1,000 | ,000 | | |
| | No infiltrado | 26 | ,000 | 1,000 | | |
| ORHistologia | ductal | 455 | ,000 | ,000 | | |
| | lobellar | 14 | 1,000 | ,000 | | |
| | others | 6 | ,000 | 1,000 | | |
| edatCat50 | <=50 | 245 | 1,000 | | | |
| | >50 | 230 | ,000 | | | |
| Graudicotomico | grau 1 o 2 | 215 | ,000 | | | |
| | grau3 | 260 | 1,000 | | | |
| Ki67cat | <30 | 157 | ,000 | | | |
| | =o>30 | 318 | 1,000 | | | |
| TILS20 | <=20 | 294 | ,000 | | | |
| | >20 | 181 | 1,000 | | | |
| TNM_cat3 | Stage II | 326 | 1,000 | | | |
| | Stage III | 149 | ,000 | | | |

**Variables en la ecuación**

| | | B | Error estándar | Wald | gl | Sig. | Exp(B) | 95% C.I. para EXP(B) Inferior | 95% C.I. para EXP(B) Superior |
|---|---|---|---|---|---|---|---|---|---|
| Paso 1[a] | TNM_cat3(1) | ,329 | ,280 | 1,388 | 1 | ,239 | 1,390 | ,804 | 2,405 |
| | Graudicotomico(1) | ,641 | ,326 | 3,870 | 1 | ,049 | 1,899 | 1,002 | 3,599 |
| | ORHistologia | | | ,000 | 2 | 1,000 | | | |
| | ORHistologia(1) | -18,752 | 10243,430 | ,000 | 1 | ,999 | ,000 | ,000 | . |
| | ORHistologia(2) | -19,105 | 15460,497 | ,000 | 1 | ,999 | ,000 | ,000 | . |
| | Molecular_sutipo | | | 28,538 | 4 | ,000 | | | |
| | Molecular_sutipo(1) | -,803 | 1,107 | ,526 | 1 | ,468 | ,448 | ,051 | 3,925 |
| | Molecular_sutipo(2) | 1,220 | ,392 | 9,698 | 1 | ,002 | 3,386 | 1,572 | 7,296 |
| | Molecular_sutipo(3) | 1,919 | ,403 | 22,660 | 1 | ,000 | 6,815 | 3,093 | 15,020 |
| | Molecular_sutipo(4) | ,711 | ,378 | 3,540 | 1 | ,060 | 2,037 | ,971 | 4,273 |
| | KI_67_cat(1) | ,743 | ,369 | 4,061 | 1 | ,044 | 2,102 | 1,021 | 4,328 |
| | TILs20(1) | -,003 | ,376 | ,000 | 1 | ,993 | ,997 | ,477 | 2,082 |
| | Tipus_infiltrat | | | 1,407 | 3 | ,704 | | | |
| | Tipus_infiltrat(1) | -,305 | ,405 | ,567 | 1 | ,451 | ,737 | ,333 | 1,631 |
| | Tipus_infiltrat(2) | ,141 | ,304 | ,215 | 1 | ,643 | 1,152 | ,634 | 2,090 |
| | Tipus_infiltrat(3) | -,061 | ,852 | ,005 | 1 | ,942 | ,940 | ,177 | 4,994 |
| | estromal_intraepitelial_cat3 | | | 2,064 | 1 | ,151 | | | |
| | estromal_intraepitelial_cat3 (1) | ,526 | ,366 | 2,064 | 1 | ,151 | 1,692 | ,826 | 3,465 |
| | Homogeneitat_TILs | | | 10,631 | 2 | ,005 | | | |
| | Homogeneitat_TILs(1) | 1,044 | ,348 | 9,024 | 1 | ,003 | 2,840 | 1,437 | 5,613 |
| | Homogeneitat_TILs(2) | -,508 | ,411 | 1,526 | 1 | ,217 | ,602 | ,269 | 1,347 |
| | Constante | -3,386 | ,488 | 48,099 | 1 | ,000 | ,034 | | |

a. Variables especificadas en el paso 1: Homogeneitat_TILs.
